# Ultrasonic Auxiliary Ozone Oxidation-Extraction Desulfurization: A Highly Efficient and Stable Process for Ultra-Deep Desulfurization

**DOI:** 10.3390/molecules27227889

**Published:** 2022-11-15

**Authors:** Rui Wang, Kaiqing Zhang, Ivan V. Kozhevnikov

**Affiliations:** 1 School of Environmental Science & Engineering, Shandong University, Qingdao 266237, China; 2 Department of Chemistry, The University of Liverpool, Liverpool L69 7ZD, UK

**Keywords:** ultra-deep desulfurization, heteropoly compound, dibenzothiophene

## Abstract

For ultra-deep desulfurization of diesel fuel, this study applied the ultrasound-assisted catalytic ozonation process to the dibenzothiophene (DBT) removal process with four Keggin-type heteropolyacids (HPA) as catalysts and acetonitrile as extractant. Through experimental evaluations, H_3_PMo_12_O_40_ was found to be the most effective catalyst for the oxidative removal of DBT. Under favorable operating conditions with a temperature of 0 °C, H_3_PMo_12_O_40_ dosage of 2.5 wt.% of *n*-octane, and ultrasonic irradiation, DBT can be effectively removed from simulated diesel. Moreover, the reused catalyst exhibited good catalytic activity in recovery experiments. This desulfurization process has high potential for ultra-deep desulfurization of diesel.

## 1. Introduction

Ultra-deep desulfurization from transportation fuels has become an increasingly important subject worldwide, due to urgent environmental problems and increasingly stringent regulations. Conventional catalytic hydrodesulfurization (HDS) is highly efficient in removing aliphatic and acyclic sulfur compounds, but is limited to reducing refractory sulfur-containing compounds such as dibenzothiophene (DBT) and its derivatives to ultra-low levels [1]. Therefore, it is urgent to develop alternative non-HDS methods to achieve clean fuels with extremely low sulfur concentrations.

One of the most promising alternative methods is oxidative desulfurization (ODS) combined with the extraction process. Compared with conventional HDS, refractory sulfur compounds can be removed under mild conditions in ODS [2]. Aqueous H_2_O_2_ is the commonly used oxidant [3]. However, the catalyzed decomposition of hydrogen peroxide may compete with sulfur-containing compound oxidation in the catalytic oxidative desulfurization process, which would cause the consumption of a huge amount of hydrogen peroxide [4].

Ozone, due to its high oxidation potential (2.07 V in acid solution) [5] and environmentally friendly nature, is widely applied in water treatment as well as in other fields [6,7]. Although ozone is a green catalyst with strong oxidizing properties, this catalyst is unstable [8]. Ozone is poorly soluble in oil products. Its oxidation effect is not satisfactory when the ozone concentration is too low [9]. Too high a level of ozone concentration is harmful to the environment and causes a drop in octane number affecting oil quality. Therefore, the proper ozone concentration should be selected in the ODS system. Heteropoly compound, an increasingly important class of environmentally friendly catalysts for various organic reactions [10], has recently attracted considerable attention. Shatalo et al. [11,12] found that molybdo-vanado-phosphate heteropolyanion catalyzing pulp ozonation in acetone/water solution was a particularly effective and selective environmentally benign bleaching approach. Therefore, it is possible that catalytic ozonation by Keggin heteropolyacids may show potential benefits in desulfurization.

Recent studies of catalytic desulfurization with Keggin heteropolyacids have focused on the preparation of loaded catalysts to improve catalytic efficiency. For example, Craven et al. [13] prepared POM/RPN-SiO_2_ catalysts by immobilizing POM (PMo, PW, and SiW) on RPN-SiO_2_ carriers using the corresponding heteropolyacids as precursors. The catalytic efficiencies of PMo, PW, and SiW in DBT oxidation were 100%, 70%, and 55%, respectively, after 3 h of catalytic reaction using 30% H_2_O_2_ as the oxidant. Rafiee et al. [14] incorporated three heteropolyacids, including H_3_PMo_12_O_40_ (PMo), H_3_PW_12_O_40_ (PW), and H_4_SiW_12_O_40_ (SiW), into HKUST-1 used for the catalytic oxidation of sulfides. The experimental results showed that the catalytic efficiency of POMS for sulfide decreases in the order of PMo > PW > SiW. Mesoporous LaVO_4_ for deep desulfurization was prepared by the hydrothermal method by Hussain et al. [15]. A photocatalytic approach was taken to remove organic sulfur compounds from diesel and this catalyst had good desulfurization efficiency under mild reaction conditions. Li et al. [16] selected MIL-101 (Al) and MIL-101 (Fe) for the immobilization of the active ingredient H_3_PMo_6_W_6_O_40_, and such catalysts had extremely high catalytic efficiency in the oxidation of DBT. Since then, Li et al. [17] have synthesized a POM-modified catalyst (POM-MIL-101F@Fibercloth), which has an excellent degradation rate for DBT under the condition of ethanol as solvent. However, there are few studies on catalytic ozone desulfurization and ultrasound-assisted desulfurization.

Based on the previous study of some metal salts of Keggin-type heteropolyacids as oxidative desulfurization catalysts by our group [18], four different types of Keggin-type heteropolyacids (HPA) were used as catalysts, and acetonitrile was used as an extractant for DBT removal. Main factors affecting the desulfurization process, including temperature, catalyst dosage, initial sulfur content, and ultrasonic irradiation, were investigated, and DBT was found to be removed effectively by this system under optimized conditions. Moreover, the recovered catalyst exhibited good catalytic activity.

## 2. Experimental Section

### 2.1. Materials

All reagents and solvents were available commercially and used without further purification, unless indicated otherwise. DBT (C_12_H_8_S, 99%) was purchased from Sigma-Aldrich. BT (C_8_H_6_S, 98%) and 4,6-DMDBT (C_14_H_12_S, 97%) were purchased from J & K Chemical Ltd. (Beijing, China). AR-grade phosphomolybdic acid (H_3_PMo_12_O_40_·xH_2_O), phosphotungstic acid (H_3_PW_12_O_40_·xH_2_O), and silicotungstic acid (H_4_SiW_12_O_40_·xH_2_O) were purchased from the National Drug and Chemical Group Co., Ltd. (Tianjin, China). The solution of DBT in *n*-octane was used as simulated diesel, in which the sulfur content was set by fixing the dosage of DBT. Ozone was produced from pure oxygen using an ozone generator.

### 2.2. Catalyst Preparation

H_3_PW_12_O_40_·xH_2_O, H_3_PMo_12_O_40_·xH_2_O and H_4_SiW_12_O_40_·xH_2_O were pretreated according to literature [19,20] to obtain H_3_PW_12_O_40_·6H_2_O, H_3_PMo_12_O_40_·13H_2_O, and H_4_SiW_12_O_40_·6H_2_O.

H_4_GeW_12_O_40_ was prepared according to the method given by Wu [21]. Germanium powder (2.625 g) was suspended in 15 mL of NaOH solution (6.25 mol/L). An aqueous H_2_O_2_ solution (~10 mol/L) was dropped slowly into the above mixture, stirring until complete metal dissolution. The obtained solution was moved into a water bath at 80 °C to decompose the hyperoxide until no further O_2_ evolution, then diluted into 100 mL, thus a germanate stock solution was obtained.

125 mL of the aqueous solution of Na_2_WO_4_ (1.25 mol/L) was mixed with 35 mL of germanate stock solution and the mixture was warmed up to 80 °C. The pH of the mixture solution was adjusted to 0.5 using concentrated aqueous HNO_3_. After the reaction proceeded for 1 h at 80 °C, the solution was cooled to room temperature. The cooled solution was extracted with ether in a sulfuric acid medium. The extractant was dissolved with a little water and then the ether was removed by flowing dry air. The remaining solution was kept in a vacuum desiccator until crystallization. The yield was about 30 g.

### 2.3. Catalyst Characterization

The properties of H_4_GeW_12_O_40_ prepared were characterized by FT-IR, TG-DSC, XPS, XRD and BET. Fourier transform infrared (FT-IR) spectra were recorded on a 5DXC IR spectrometer in the wave number interval between 4000 and 400 cm^−1^ with a 2 cm^−1^ resolution, and samples were measured with KBr pellets. TG-DSC was performed on an SDT Q600 Universal V4.1D TA instrument operating under a nitrogen flow of 20 mL/min, at a 10 °C/min heating rate up to 700 °C and using 25–50 mg sample. X-ray diffraction (XRD) patterns were collected by a PAN alytical X-pert 3 instrument using a CuKα-ray source (λ = 0.154 nm, 40 mA × 40 kV) with a scanning speed of 15° min^−1^. The elemental composition was tested by an ESCALAB 250Xi X-ray energy spectrometer. The specific surface area and porosity of the materials were measured by Micromeritics ASAP 2460.

### 2.4. Experimental Method

**Figure 1:**
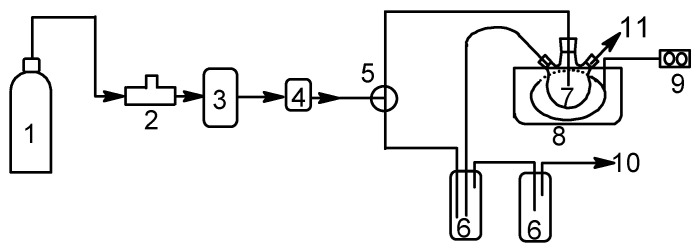
Diagram of the experimental setup. (1) oxygen cylinder; (2) mass flow controller; (3) ozone generator; (4) ozone monitor; (5) three-way valve; (6) KI traps (7) ozone reactor; (8) ultrasonic generator; (9) temperature control system; (10) gas outlet; (11) sampling.

A schematic diagram of the ozonation system is shown in Figure 1. A three-necked 250 mL round-bottomed flask was used as an ozonation reactor for the desulfurization experiments. The middle neck connected with a gas disperser through which ozone was introduced into the reaction solution at a constant rate. One of the two side necks connected with a water-cooled reflux condenser to prevent reaction solution loss and to serve as a gas outlet, and the residual ozone in the outlet gas was adsorbed by KI solution. The other was closed with a glass stopper. After 60 mL of simulated diesel and 60 mL of acetonitrile were added to the flask, the catalyst was added to the above mixture, and then ozone was bubbled up into the mixture. Sonication was performed with a KQ-100 KDB ultrasonic generator (100 W, 20 kHz, Kunshan Ultrasonic Instrument Co., Ltd., Suzhou, China). The flask was immersed into the ultrasonic batch, inside which a temperature control system was placed to keep a certain temperature, and a low temperature was achieved by adding ice to the ultrasonic batch. During the reactions, the upper *n*-octane phase of the reaction mixture was periodically withdrawn and determined for sulfur content using a Model WK-2E microcoulometric integrated analyzer (Jiangsu Jiang Fen Electroanalytical Instrument Co., Ltd., Taizhou, China).

## 3. Discussion

### 3.1. Characterizations of the Catalysts

**Figure 2:**
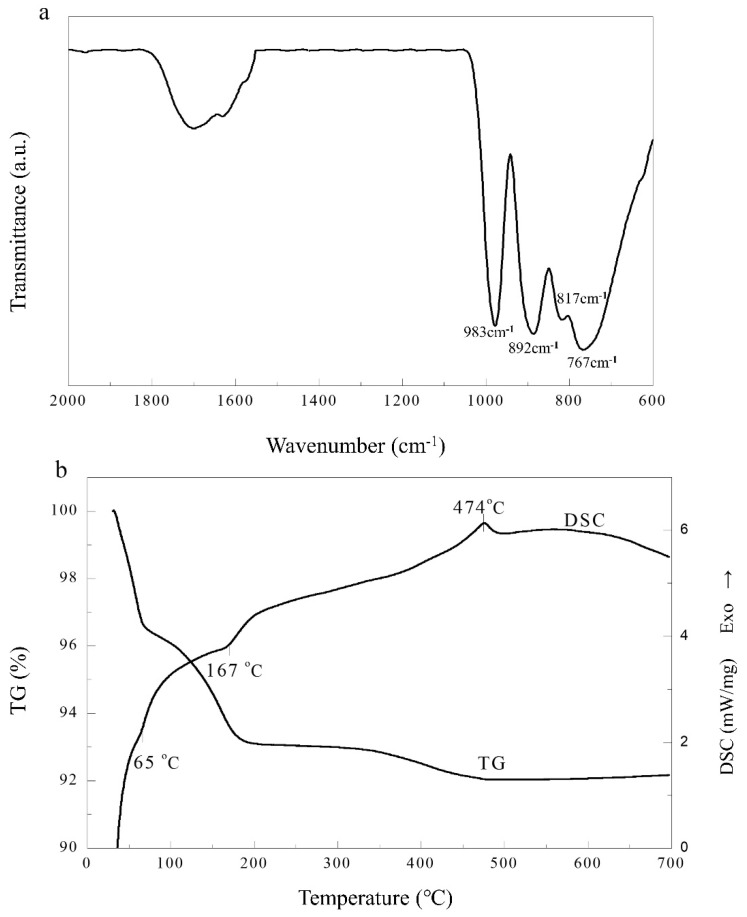
(**a**) FT-IR spectra of the H_4_GeW_12_O_40_. (**b**) TG–DSC curves of H_4_GeW_12_O_40_.

Characteristic vibration bands of Keggin structure appear in the region of 700 cm^−1^ to 1100 cm^−1^ [22]. As shown in Figure 2a, the IR spectrum of H_4_GeW_12_O_40_ shows strong vibration bands at 983, 892, 817, and 767 cm^−1^, which correspond respectively to the asymmetric vibrations W-Od (terminal O bonded to W), W-Ob (edge-sharing O connecting W), Ge-Oa (internal O atom connecting Ge and W), and W-Oc (corner-sharing O connecting W_3_O_13_ units) [23]. Hence, the desired 12-tungstogermanic acid with Keggin structure can be confirmed according to the presence of these adsorption peaks.

The thermal behavior of the hydrated H_4_GeW_12_O_40_ was investigated by TG-DSC, and the results are shown in Figure 2b. There were three steps of weight loss in the TG curve. Before 70 °C and between 70–202 °C, the mass losses were both 3.45%, which demonstrated that six molecules of zeolite water and six molecules of protonized water were lost [19,24]. The DSC curve showed two corresponding endothermal peaks at 65 and 167 °C due to the release of zeolite water and protonized water, respectively. Above 202 °C, the slow weight loss of 1.06% in the TG curve can be attributed to the departure of two molecules of structural water, and an exothermal peak centered at 474 °C in the DSC curve was due to the decomposition of H_4_GeW_12_O_40_, consistent with the result studied by Wang et al. [20]. Thus, the formula of tungstogermanic heteropoly acids synthesized in this paper was H_4_GeW_12_O_40_·12H_2_O.

**Figure 3:**
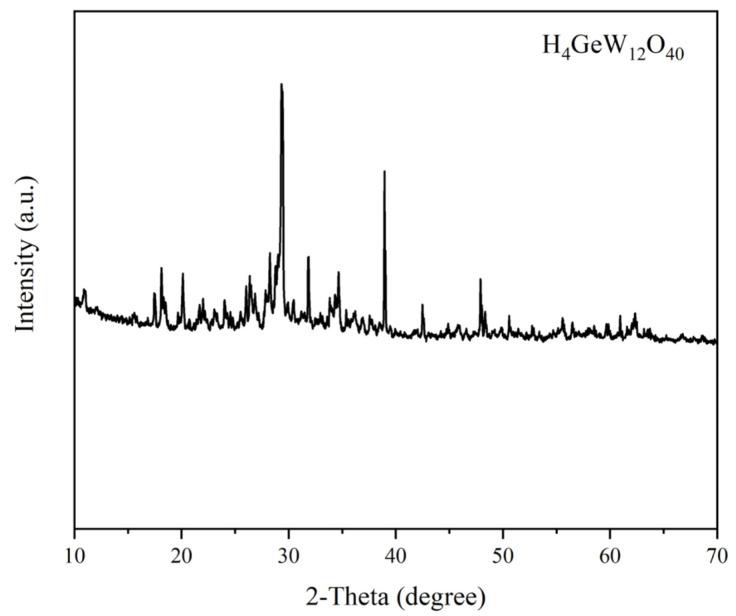
XRD patterns of prepared H_4_GeW_12_O_40_.

The XRD pattern of H_4_GeW_12_O_40_ is shown in Figure 3. The curves showed strong characteristic diffraction peaks in the ranges of 15–23°, 26–33° and 36–39°, indicating that H_4_GeW_12_O_40_ has a Keggin-type structure, which is consistent with reports in the literature [25]. This indicates the successful synthesis of H_4_GeW_12_O_40_.

**Figure 4:**
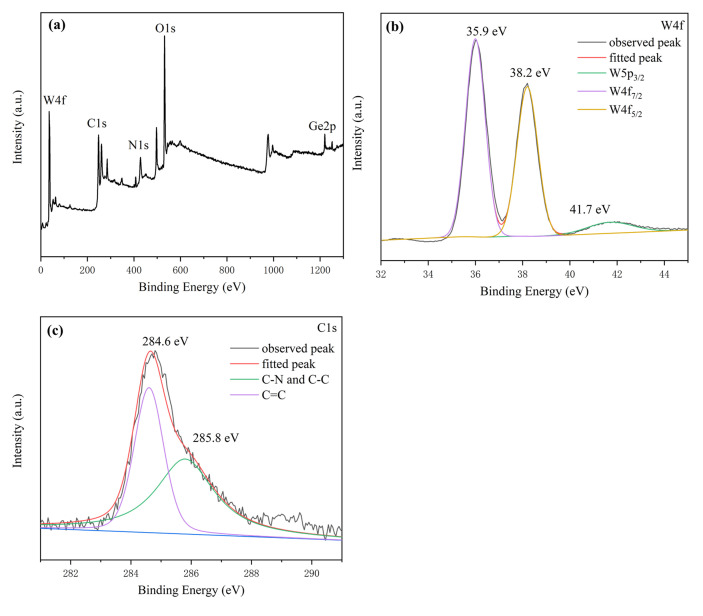
XPS spectra of H_4_GeW_12_O_40_ ((**a**) survey spectrum; (**b**) W; (**c**) C.).

The XPS spectra of H_4_GeW_12_O_40_ are shown in Figure 4, and the high-resolution spectra of W 4f and C 1s were fitted. The W4f high-resolution spectra were fitted with three typical peaks at 35.9 eV, 38.2 eV, and 41.7 eV, corresponding to W 4f_7/2_, W 4f_5/2_, and W 5p_3/2_, respectively. Two single peaks exist in the C 1s high-resolution spectra, corresponding to C=C (284.6 eV), C-N, and C-C (285.8 eV). The measured results are essentially the same as the peak positions of the XPS spectra of [GeW_12_O_40_]^4−^ anion in other literature [26], further suggesting that H_4_GeW_12_O_40_ has a Keggin-type structure.

**Figure 5:**
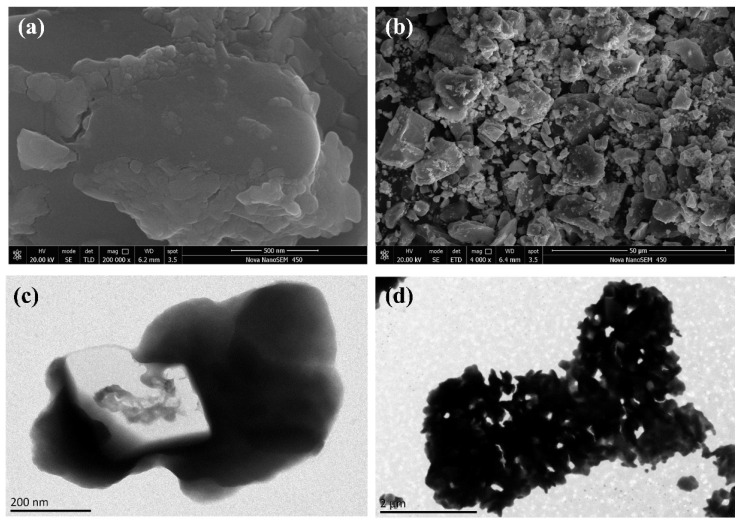
SEM images of H_4_GeW_12_O_40_ (**a**,**b**) and the TEM images of H_4_GeW_12_O_40_ (**c**,**d**).

The morphology of the synthesized material was studied using SEM images (Figure 5a,b). Irregularly shaped structures can be found on the H_4_GeW_12_O_40_ compound, and the size of the structures varies between 50 and 300 nm. The surface was found to contain a large number of small coarse particles at high magnification, which is also consistent with the surface morphological features described in the literature [27]. To further analyze the surface morphology, TEM images of c are shown in Figure 5c,d. Similar to the SEM results, Figure 5c shows the overall morphology of the catalyst similar to the SEM results. In Figure 5d, the morphology of the surface particles can be observed at higher magnification. Further BET tests were performed on the synthesized catalysts, and the surface area, pore volume, and pore size of H_4_GeW_12_O_40_ are shown in Table 1.

### 3.2. Catalytic Performance Evaluation

The catalytic activity of the Keggin-type HPAs on the oxidation removal of DBT was evaluated according to the experiments carried out at 35 °C for 60 min accompanied with ultrasound irradiation, using the above-mentioned experiment system with an initial sulfur concentration of 500 ppm, ozone dosage of 0.1 g/h, and a catalyst amount of 2.5 wt.% of *n*-octane. The results are shown in Table 1.

It can be observed from Table 2 that Keggin-type HPAs were effective in catalyzing ozone oxidation of DBT in simulated diesel. H_3_PMo_12_O_40_ exhibited the best catalytic performance with a desulfurization efficiency of 93.3%, and the other W-containing HPAs were inferior to it. The catalytic performance of the HPAs with Mo as polyatom was superior to those with W as polyatom. This result may be related to the oxidative ability of HPAs in that the oxidation capacity of Mo-containing heteropolyanions is higher than that of W-containing heteropolyanions [28,29].

Among the catalyst HPAs with W as polyatom, the catalytic activity of H_3_PW_12_O_40_ was the highest, reaching 89.3% of removal efficiency in 60 min. The removal of DBT catalyzed by H_4_GeW_12_O_40_ and SiW_12_O_40_^4−^ was 86.4% and 85.7%, respectively. The catalytic activities of these catalysts increased in the order of H_4_SiW_12_O_40_ < H_4_GeW_12_O_40_ < H_3_PW_12_O_40_, which agreed fairly well with the order of the oxidation potential of the polyanions, SiW_12_O_40_^4−^ < GeW_12_O_40_^4−^ < PW_12_O_40_ [3,4,5,6,7,10,11,19,20,21,22,23]. These results suggested that oxidative desulfurization was mainly affected by the oxidizing ability of HPAs.

Based on the results, it can be concluded that oxidizing ability of HPAs played a significant role in the ultrasound-assisted catalytic ozone oxidation process for desulfurization, and H_3_PMo_12_O_40_ (HPMo) was chosen for additional experiments on the effects of several operational factors.

### 3.3. Influence of Reaction Conditions

#### 3.3.1. Effect of Temperature

**Figure 6:**
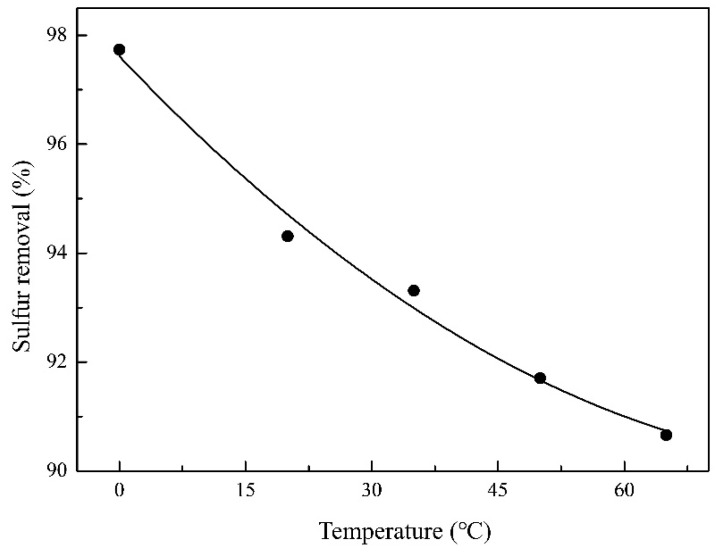
The effect of temperature on DBT removal. Experimental conditions: initial sulfur concentration, 500 ppm; catalyst dosage, 2.5 wt.% of *n*-octane; ozone dosage, 0.1 g/h; reaction time, 60 min.

Temperature has a significant effect on DBT removal. From the results shown in Figure 6, it was evident that desulfurization efficiency decreased with the increase in temperature. At lower temperatures, such as at 0 °C, the desulfurization efficiency was 98.1% for a 60 min reaction, while with the temperature increased to 65 °C, the desulfurization efficiency decreased to 90.7%. The increase in temperature can influence the catalytic ozonation process in two ways: (1) the concentration of ozone in solution is reduced; (2) the diffusion rate of the reacting substances is enhanced. The efficiency decrease in DBT removal from 0 to 65 °C may demonstrate that (1) is predominant. Therefore, 0 °C can be recommended as the ideal temperature in this paper.

#### 3.3.2. Effect of Catalyst Dosage

**Figure 7:**
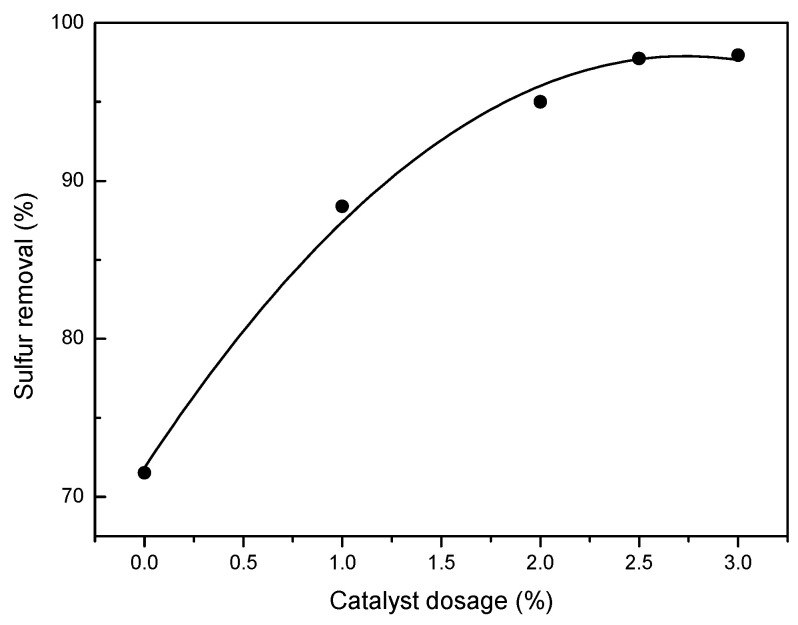
The effect of catalyst dosage on DBT removal. Experimental conditions: initial sulfur concentration, 500 ppm; temperature, 0 °C; ozone dosage, 0.1 g/h; reaction time, 60 min.

Catalyst dosage also plays an important role in the oxidation of DBT. The results are presented in Figure 7. Under otherwise identical conditions, without catalyst, 71.5% of the DBT was removed from the *n*-octane phase in 60 min by the joint action of extraction and direct ozonation with ultrasonic irradiation. The efficiencies of DBT removal in the presence of HPMo increased remarkably from 88.4% to 98.1% with increasing weight percent of HPMo over the whole solution from 1.0 wt.% to 2.5 wt.%, and then leveled off from 2.5 wt.% to 3.0 wt.%. From the results, a suitable catalyst amount can be identified as 2.5 wt.% of the *n*-octane.

#### 3.3.3. Effect of Initial Sulfur Concentration

**Figure 8:**
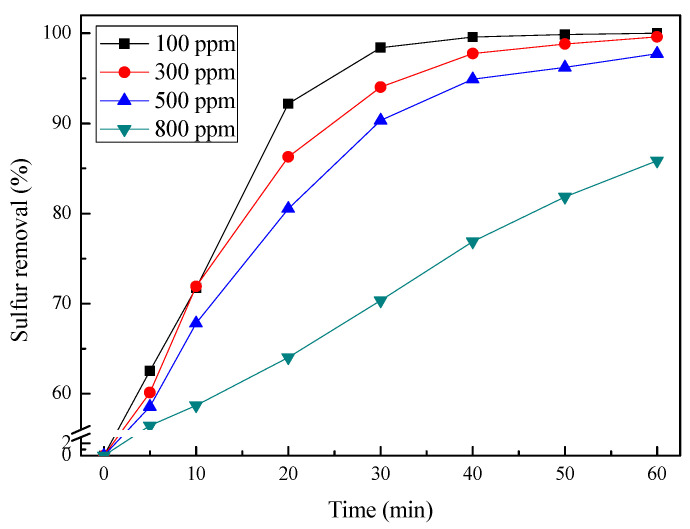
The effect of initial sulfur concentration on DBT removal. Experimental conditions: catalyst dosage, 2.5 wt.% of *n*-octane; temperature, 0 °C; ozone dosage, 0.1 g/h.

Another significant factor for sulfur removal is the initial sulfur concentration, which was investigated using different concentrations (100, 300, 500, and 800 ppm). The results are shown in Figure 8. An increase in the initial sulfur concentration led to a remarkable decrease in the removal of DBT. When the initial sulfur concentration was 100 ppm, the conversion of DBT was up to 100% in 60 min, then as initial sulfur concentration increased to 300 ppm, 500 ppm, and further to 800 ppm, the desulfurization efficiency was found to decrease from 99.6% to 98.1%, and further to 85.9%, corresponding to 1.2 ppm, 10.0 ppm, and 112.8 ppm, respectively. This phenomenon indicated that DBT removal depends on its initial concentration, which may be caused by the decrease in catalytic active sites. As the initial concentration of DBT increased, more DBT molecules were absorbed into the catalyst. Thus, an increase in the amount of substrates accommodating the catalyst inhibits the action of the catalyst with O_3_. Therefore, the desulfurization efficiency decreased.

#### 3.3.4. Effect of Ultrasonic Irradiation

**Figure 9:**
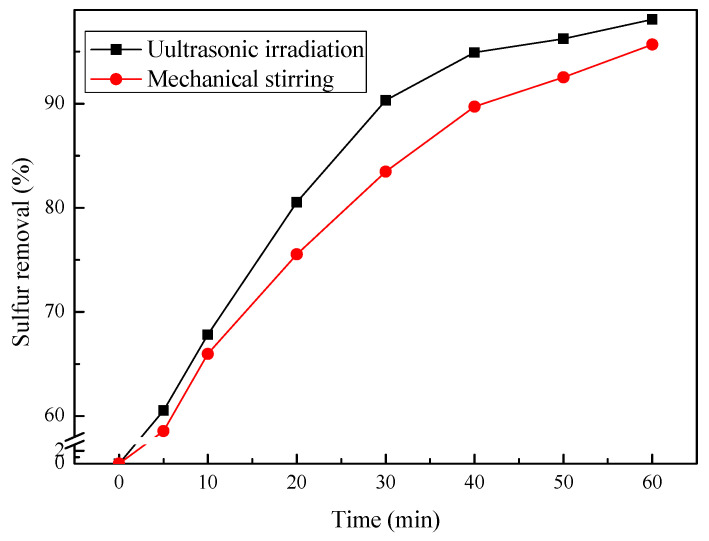
The effect of ultrasound on DBT removal. Experimental conditions: temperature, 0 °C; initial sulfur concentration, 500 ppm; catalyst dosage, 2.5 wt.% of *n*-octane; ozone dosage, 0.1 g/h; reaction time, 60 min.

Ultrasonic irradiation can significantly enhance the reaction efficiency in chemical synthesis [30], since cavitation can be produced when mechanical vibrations are transmitted into the liquid as ultrasonic waves. In order to evaluate the effect of ultrasonic waves on the removal of DBT, a procedure applying the same previously optimized conditions was conducted, with a temperature of 0 °C, catalyst dosage of 2.5 wt.% of *n*-octane, and initial sulfur concentration of 500 ppm. However, mechanical stirring was employed instead of ultrasonic irradiation. The results are shown in Figure 9.

The removal of DBT in catalytic ozonation with mechanical stirring was 94.5% for 60 min reaction. When ultrasonic irradiation was applied in catalytic ozonation, the removal of DBT increased to 98.1%. The efficiency of DBT removal with ultrasonic irradiation at different intervals evaluated were all higher than those with mechanical stirring. These results indicated that ultrasound enhances the mass transfer of ozone from the gas phase to the reaction solution and accelerates the whole desulfurization process. In this way, a better desulfurization effect is achieved.

#### 3.3.5. Effect of Sulfide Species

**Figure 10:**
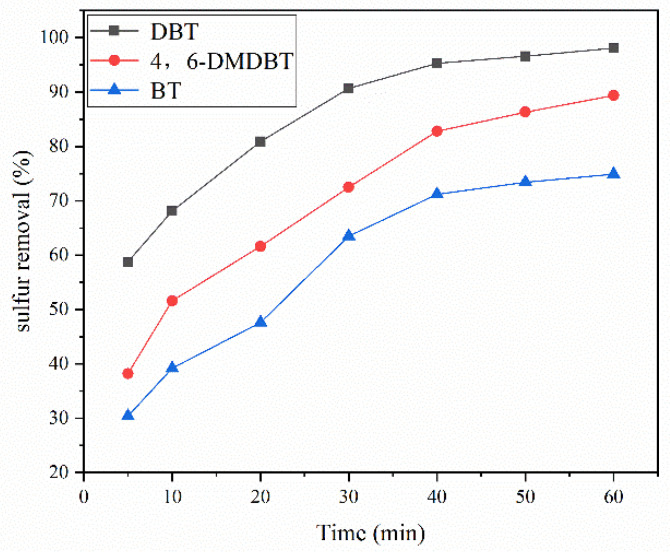
HPMo removal efficiency for different sulfides. Experimental conditions: temperature, 0 °C; initial sulfur concentration, 500 ppm; catalyst dosage, 2.5 wt.% of *n*-octane; ozone dosage, 0.1 g/h; reaction time, 60 min.

To investigate the removal effect of HPMo on different sulfides, simulated diesel fuel with 500 ppm sulfur content of DBT, 4,6-DMDBT, and BT were configured to investigate the selectivity of the ODS system for different sulfides. As shown in Figure 10, the removal efficiencies of the sulfur compounds in the HPMo catalyst system were DBT > 4,6-DMDBT > BT. The reactivity of the thiophene sulfides was positively correlated with the electron cloud density of the sulfur atoms, which were 5.758, 5.760, and 5.739 for DBT, 4,6-DMDBT, and BT, respectively [31]. The low electron cloud density of the sulfur atoms of BT made it the most difficult of the three sulfides to remove, followed by 4,6-DMDBT, which was more difficult to remove than DBT, probably due to the greater steric effects of 4,6-DMDBT on the molecular methyl group.

#### 3.3.6. Catalyst Reuse

**Figure 11:**
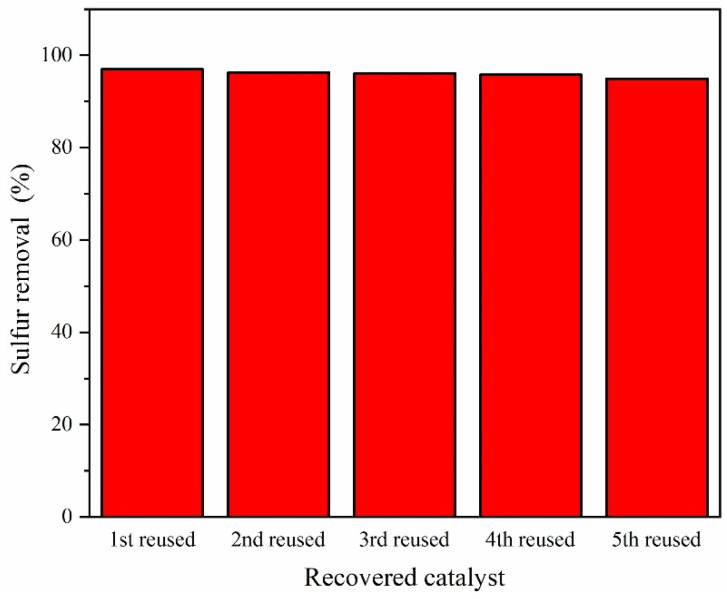
The ODS performance of the reused catalyst.

The catalytic effect of the used catalyst was explored and the recovering process was performed according to the method reported by Zhang et al. [32]. After completion of the reaction, the reaction mixture was kept still for 20 min. Then, the acetonitrile phase including the catalyst was separated and distilled to recover acetonitrile by cooling at the top of the distillation column. Water was added to the residue in the evaporator, and then, adding diethyl ether to the above mixture, HPMo was extracted into the diethyl ether phase. HPMo was recovered by further evaporation of diethyl ether. Evaluated through five recovery-reusing runs, the recovered HPMo catalyst was found to demonstrate excellent catalytic performance (Figure 11). The reused catalysts achieved 97%, 96.3%, 96.1%, 95.8%, and 94.9% DBT conversions in the first, second, third, fourth and fifth ODS cycles, respectively.

## 4. Conclusions

Ultrasound-assisted catalytic ozone oxidation process exhibited high efficiency for the removal of DBT from simulated diesel. The catalytic performance of Keggin-type HPAs was mainly related to the oxidizing ability of HPAs and followed the order of H_3_PMo_12_O_40_ > H_3_PW_12_O_40_ > H_4_SiW_12_O_40_ > H_4_GeW_12_O_40_ in the desulfurization process. Factors affecting the ODS process were investigated, including temperature, catalyst dosage, initial sulfur content, and ultrasound irradiation, whereby the favorable operating conditions were recommended as reaction temperature, 0 °C; catalyst dosage, 2.5 wt.% of *n*-octane; and ultrasound irradiation. DBT removal depended on its initial concentration, and increasing initial sulfur concentration led to a decrease in desulfurization efficiency. Furthermore, the used catalyst was recoverable and demonstrated excellent catalytic performance. As a whole, the ultrasound-assisted catalytic ozone oxidation process has a good application prospect to obtain ultra-low sulfur diesel.

## Figures and Tables

**Table 1:** BET measurements of the as-synthesized Catalyst.

Catalyst	Surface Area [m^2^/ g]	Surface Area BJH_Ads_ [m^2^/ g]	Surface Area BJH_Des_ [m^2^/g]	Pore Size BJH_Ads_ [nm]
H_4_GeW_12_O_40_	2.21	2.57	2.09	4.9
Catalyst	Pore size BJH_Des_ [nm]	Pore volume BJH_Ads_ [cm^3^/g]	Pore volume BJH_Des_ [cm^3^/g]	
H_4_GeW_12_O_40_	6.5	0.032	0.031	

**Table 2:** Catalytic effects of HPAs on the oxidation of DBT.

Catalyst	Efficiency (%)
H_3_PW_12_O_40_	89.3
H_3_PMo_12_O_40_	93.3
H_4_SiW_12_O_40_	85.7
H_4_GeW_12_O_40_	86.4
none	73.9

## Data Availability

Not applicable.

## Sample Availability

Samples of the H_3_PMo_12_O_40_ are available from the authors.
